# Auditing the Representation of Female Versus Male Athletes in Sports Science and Sports Medicine Research: Evidence-Based Performance Supplements

**DOI:** 10.3390/nu14050953

**Published:** 2022-02-23

**Authors:** Ella S. Smith, Alannah K. A. McKay, Megan Kuikman, Kathryn E. Ackerman, Rachel Harris, Kirsty J. Elliott-Sale, Trent Stellingwerff, Louise M. Burke

**Affiliations:** 1 Mary MacKillop Institute for Health Research, Australian Catholic University, Melbourne, VIC 3000, Australia; alannah.mckay@acu.edu.au (A.K.A.M.); megan.kuikman@myacu.edu.au (M.K.); louise.burke@acu.edu.au (L.M.B.); 2 Wu Tsai Female Athlete Program, Boston Children’s Hospital and Harvard Medical School, Boston, MA 02115, USA; kathryn.ackerman@childrens.harvard.edu; 3 Female Athlete Performance and Health Initiative, Australian Institute of Sport, Canberra, ACT 2617, Australia; drrachharris@gmail.com; 4 Perth Orthopaedic and Sports Medicine Research Institute, West Perth, WA 6005, Australia; 5 Musculoskeletal Physiology Research Group, Sport Health and Performance Enhancement Research Centre, Nottingham Trent University, Nottingham NG1 4FQ, UK; kirsty.elliottsale@ntu.ac.uk; 6 Canadian Sport Institute-Pacific, Institute for Sport Excellence, 4371 Interurban Road, Victoria, BC V9E 2C5, Canada; tstellingwerff@csipacific.ca; 7 Exercise Science, Physical and Health Education, University of Victoria, Victoria, BC V8P 5C2, Canada

**Keywords:** women, physical activity, menstrual status, oral contraceptive, nutrition

## Abstract

Although sports nutrition guidelines promote evidence-based practice, it is unclear whether women have been adequately included in the underpinning research. In view of the high usage rates of performance supplements by female athletes, we conducted a standardised audit of the literature supporting evidence-based products: β-alanine, caffeine, creatine, glycerol, nitrate/beetroot juice and sodium bicarbonate. Within 1826 studies totalling 34,889 participants, just 23% of participants were women, although 34% of studies included at least one woman. Across different supplements, 0–8% of studies investigated women exclusively, while fewer (0–2%) were specifically designed to compare sex-based responses. The annual publication of female-specific studies was ~8 times fewer than those investigating exclusively male cohorts. Interestingly, 15% of the female participants were classified as international/world-class athletes, compared with 7% of men. Most studies investigated performance outcomes but displayed poorer representation of women (16% of participants), whereas health-focussed studies had the greatest proportion of female participants (35%). Only 14% of studies including women attempted to define menstrual status, with only three studies (~0.5%) implementing best practice methodologies to assess menstrual status. New research should target the efficacy of performance supplements in female athletes, and future sports nutrition recommendations should specifically consider how well female athletes have contributed to the evidence-base.

## 1. Introduction

Female-targeted research related to sports science/sports medicine (SSSM) has failed to mirror the increase in participation and popularity of women’s sport [1]. While many aspects of women’s sport gain parity with their male counterparts, audits show a substantial under-representation of women as study participants, with female-only projects accounting for just 4–13% of all studies [2,3,4,5,6]. Of the multiple explanations [1], the complexity of female physiology represents a long-standing challenge, with the additional intricacy, time and expense involved in study designs being a deterrent to many researchers. The practical result of female under-representation in SSSM research is that most guidelines for training and nutrition strategies to optimise performance are underpinned by research conducted in men, without consideration of issues associated with the application to female athletes. This lack of robust research on female-specific considerations ultimately hinders the SSSM practitioner in implementing an evidence-based approach.

Multiple biological and phenotypical sex differences influence biomechanics, metabolism, hydration, thermoregulation, fatigue and, ultimately, sports performance [7,8,9,10,11,12]. Moreover, sport characteristics or playing styles may differ between the sexes (e.g., shorter distances/durations or lighter equipment for women) [13,14]. It is therefore problematic to apply conclusions from male-based studies directly to women without considering the potential influences of sexual dimorphisms or event-specific demands. Indeed, the gold standard of evidence-based practice is hindered by concerns over the ecological validity and transferability of findings from studies of male athletes in the absence of robust research on female-specific considerations. A tactical approach to redressing this situation includes auditing the current literature [1] to identify themes that could be considered high priority. These include issues for which there is minimal information on female-specific response to an intervention; a potential difference/unique sex-related consideration; a popular interest or usage in the strategy; and/or the likelihood of a substantial magnitude of change associated with optimal practice.

The use of performance supplements [15] intersects with many of these characteristics. Surveys of high-level (national to Olympic) athletes typically report that 80–90% are supplement users, with women reporting a slightly higher prevalence of use when sex-specific patterns are reported [16]. A small number of products that can directly enhance sports performance via identifiable mechanisms with meaningful (e.g., 2–3%) margins of improvement have been identified [17]. However, it is unclear whether female athletes have been specifically considered in the development of the current best-practice supplementation protocols. Therefore, our aim was to review the current literature, utilising our recently developed audit protocol [1], to examine the representation of women in studies that support recognised performance supplements. We chose the six performance supplements included in Category A of the Australian Institute of Sport Sports Supplement Framework [18] (β-alanine, caffeine, creatine, glycerol, nitrate/beetroot juice and sodium bicarbonate), described as “supplements with strong scientific evidence for use in specific situations in sport using evidence-based protocols” [15]. Our audit targeted the quality and quantity of research addressing the female high-performance athlete to identify her inclusion in literature informing current sports nutrition guidelines for supplement use.

## 2. Materials and Methods

This audit was conducted according to methods comprehensively outlined by Smith et al. [1]. Information specific to the current audit is detailed below.

### 2.1. Search Strategy

An electronic literature search of PubMed was conducted using the terms: “(athlete OR sport OR healthy) AND (** supplement specific terms **) AND (exercise OR performance OR endurance OR aerobic OR strength OR power OR anaerobic) NOT animal NOT rodent NOT diabetes NOT obese NOT overweight NOT COPD”. Supplement-specific terms were: (1) B-alanine OR beta-alanine; (2) caffeine OR energy drink OR coffee; (3) creatine OR creatine monohydrate OR creatine anhydrous OR creatine hydrochloride; (4) glycerol OR glycerine OR glycerine; (5) sodium bicarbonate OR bicarbonate of soda OR NaHCO3 OR HCO3 OR baking soda and (6) beetroot juice OR nitrate OR nitrite. Searches were exclusive to original research papers among human participants, published in English, without date restrictions and current to 1 September 2021. Following the initial search, select review articles for each supplement were screened for further relevant papers that were not detected in the primary search.

### 2.2. Data Extraction

**Figure 1:**
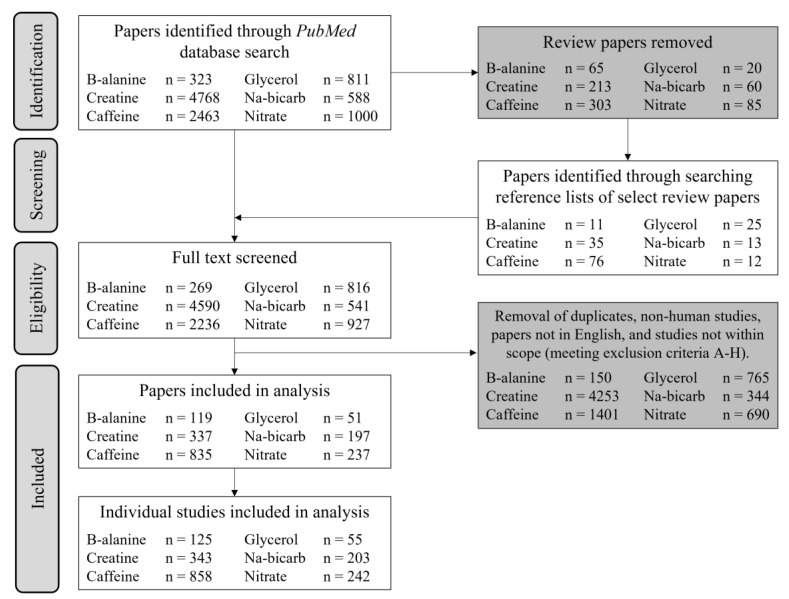
Flowchart demonstrating the primary screening process for each performance supplement audited, and the total number of individual studies included for extraction of metrics A–F.

The titles, abstracts and full texts were initially screened using Rayyan online software [19] to identify papers for inclusion (Figure 1). The outcomes of interest were direct measurements of performance or health parameters, and indirect contributors/markers of performance/health. Duplicates and reviews were removed, alongside papers meeting the exclusion criteria: (a) >50 years of age and untrained (i.e., older populations other than identified masters athletes), (b) children (sports-specific criteria), (c) presence of lifestyle diseases (e.g., obesity, hypertension) or smoking, (d) multi-ingredient products, unless the supplement of interest was the primary ingredient, (e) failure to investigate the supplement as the primary outcome/independent variable, (f) outcomes irrelevant to performance, health or indirect associations with performance/health, and (g) failure to explicitly state the sex or male: female ratio of the participants. For papers reporting several separate studies, each dataset was analysed individually. Hereafter, ‘paper’ refers to the entire publication, whereas ‘study’ describes discrete investigations within a paper.

Following the initial screening, details of the following metrics were extracted: (a) population, (b) athletic calibre [20]: Tier 0 (sedentary), Tier 1 (recreationally active), Tier 2 (trained/developmental), Tier 3 (highly trained/national), Tier 4 (elite/international) and Tier 5 (world-class), (c) menstrual status (methodological consideration graded as bronze/silver/gold standard or ungraded), (d) research theme (performance/health/indirect associations with performance or health), (e) journal publication dates and study impact (Altmetric scores on 1 September 2021) and (f) sample size (distinguishing male/female participants where applicable). The extraction protocol is described in detail elsewhere [1]. In the present audit, it was appropriate to include participants with a “tier 0” athletic calibre due to the relevance of mechanistic studies not requiring an exercise condition. Studies involving separate investigations of several supplements were included in the audit for each supplement, and their metrics counted separately.

### 2.3. Statistical Analysis

Analyses were performed using R Studio (v3.5.2) with statistical significance accepted at an α level of *p* ≤ 0.05. Frequency based metrics (A–D) were reported as a percentage of the total studies/participants examining a single supplement, with ranges provided for outcomes of each supplement audit. Since inspection of the histograms for journal impact factor (IF), Altmetric scores and male/female specific sample size (metrics E,F) revealed a positive skew, the results were reported as median ± interquartile range (IQR). A linear mixed model to account for unequal group sizes, using supplement and population as fixed effects, was conducted on metrics E and F. The proportion of studies achieving Altmetric scores >20 was assessed in a binary manner to delineate studies receiving greater attention than their contemporaries [21].

## 3. Results

**Figure 2:**
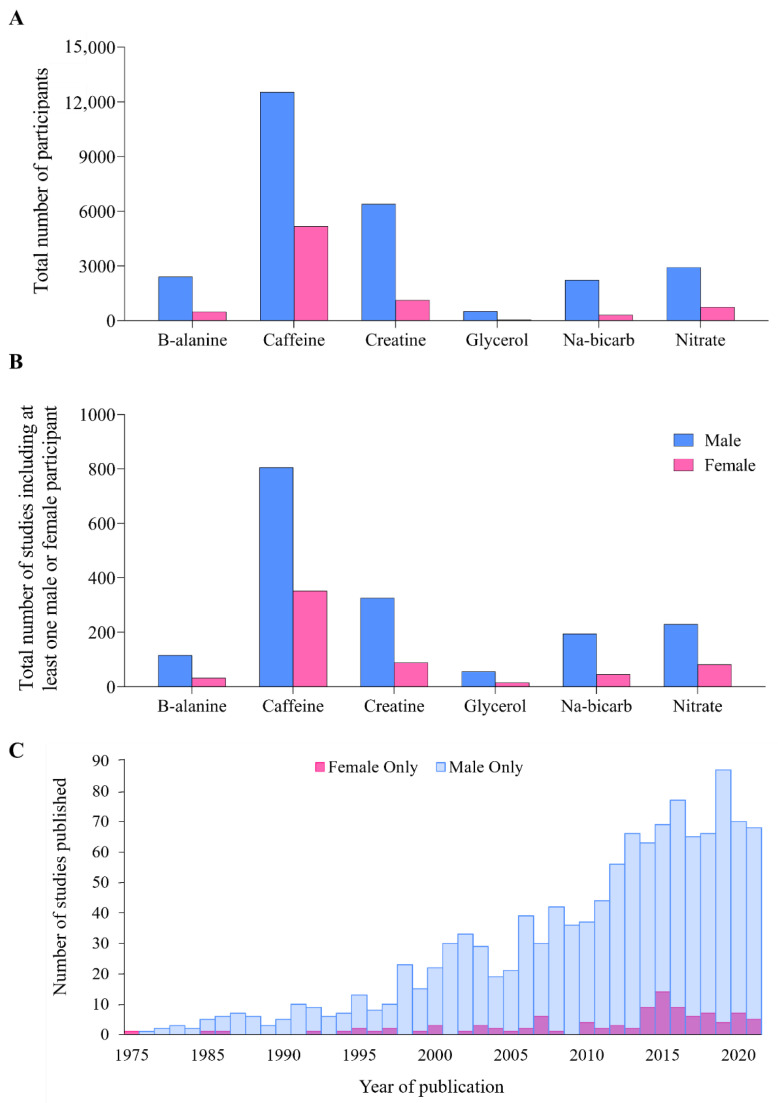
(**A**) The total number of male and female participants included across all studies, separated per supplement, (**B**) the total number of studies including at least one male or female participant, per supplement, and (**C**) histogram displaying the total number of studies published in exclusively male or female participants between 1975 and 2021 across all performance supplements.

Across the six performance supplements (β-alanine, caffeine, creatine, glycerol, nitrate/beetroot juice and sodium bicarbonate), 1826 studies totalling 34,889 participants met the inclusion criteria (Figure 1). Overall, 7884 participants were women (23%, Figure 2A) but 614 studies (34%, Figure 2B) included at least one woman. Detailed results for each supplement are included (Supplementary Material Figures S1–S6). The combined data for all six supplements revealed an annual publication of ~4 studies in which females were investigated in isolation: ~8 times fewer than the ~30 studies/year in male-only cohorts (Figure 2C). Although female-only studies increased in number over the previous eight years (Figure 2C), this reflected a general increase in the literature, with male-only studies remaining ~9 times higher than women-only cohorts.

### 3.1. Population and Sample Size

**Figure 3:**
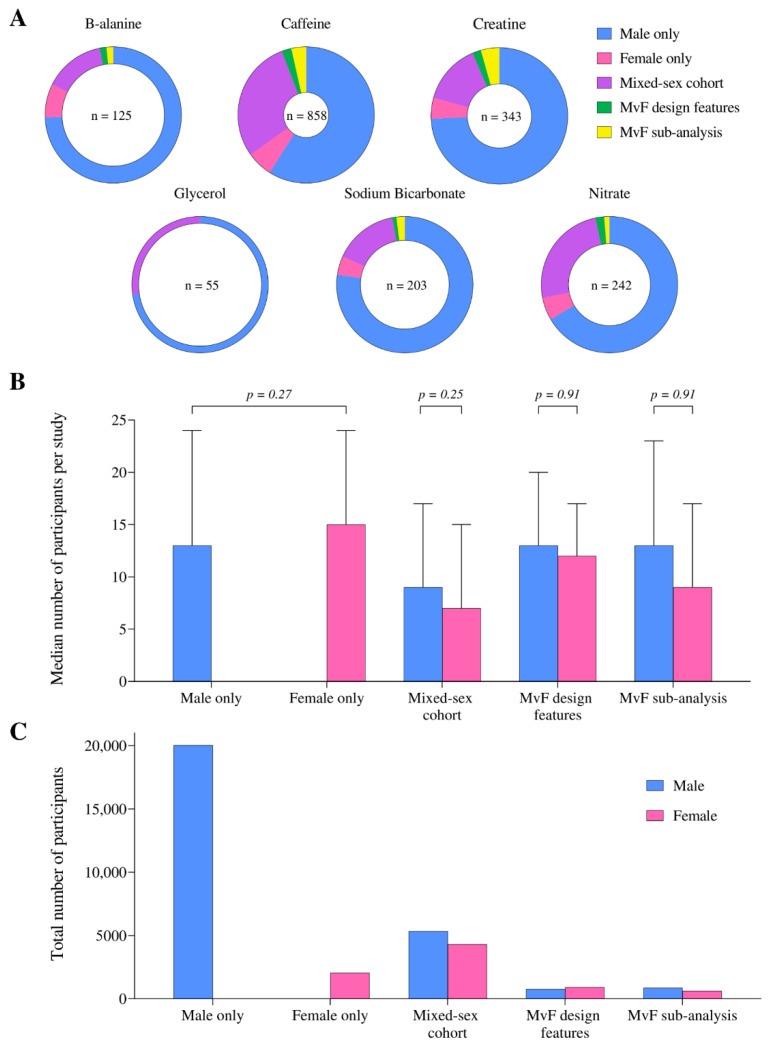
(**A**) The proportion of studies published in each population, separated by performance supplement, (**B**) the median number of male and female participants per study, and (**C**) total number of male and female participants, according to the population studied. Male versus female (MvF).

Across different supplements, 0–8% of studies investigated women exclusively, while 59–77% of studies involved male-only protocols (Figure 3A). Fewer (0–4%) conducted sex-based comparisons within the statistical procedures (male versus female (MvF) sub-analysis), with 0–2% including methodological designs specifically comparing sex-based responses (MvF design features). Most (56%) studies including women involved a mixed-sex cohort design.

There was no difference in the sample size of male- and female-only studies across all supplements (10–24 participants per study, *p* = 0.27, Figure 3B). In studies investigating both sexes (mixed-sex cohort, MvF sub-analysis and MvF design features), although men consistently accounted for a larger proportion of the cohort than women across all supplements (53–58%), this difference was not significant (all *p* > 0.05, Figure 3B). Male-specific study designs included 20,033 male participants; ~10 times more than the 2043 women studied in exclusively female cohorts (Figure 3C). Fewer (1627 men and 1536 women) participated in studies evaluating sex-differences in supplement responses (MvF sub-analysis and MvF design features).

### 3.2. Athletic Calibre

**Figure 4:**
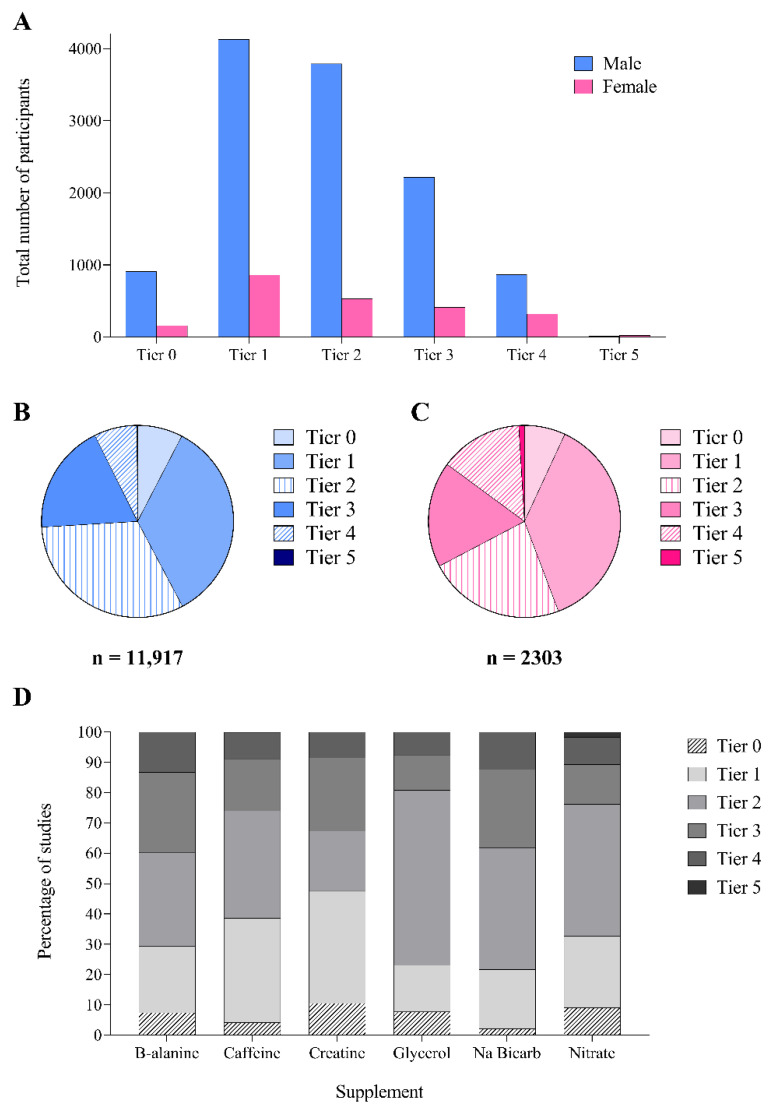
The total (**A**) and proportion (**B**,**C**) of male and female participants in each athletic tier [20]: Tier 0 (sedentary), Tier 1 (recreationally active), Tier 2 (trained/developmental), Tier 3 (highly trained/national), Tier 4 (elite/international) and Tier 5 (world-class). (**D**) The proportion of participants in each athletic tier according to the supplement investigated. Only classifiable participants are reported in all figures.

Overall, most (56%) studies failed to provide sufficient information to classify participants according to a single category of athletic calibre. Among classifiable studies, the participants were mostly from Tiers 1 (30%) and 2 (34.5%), with Tiers 0, 3, 4 and 5 accounting for 6%, 19.5%, 9.5% and 0.5% of studies, respectively (Figure 4A). Classifiable female participants were typically of higher calibre (33% from Tiers 3–5) than male participants (26% from Tiers 3–5) (Figure 4B,C). Tier 3–5 athletes participated more frequently in studies involving β-alanine (21%, *n* = 27 studies) and sodium bicarbonate (18%, *n* = 37 studies) (Figure 4D), while glycerol (9%, *n* = 5 studies) and caffeine (11%, *n* = 94 studies) involved the lowest percentage of participants from these tiers.

### 3.3. Menstrual Status

**Figure 5:**
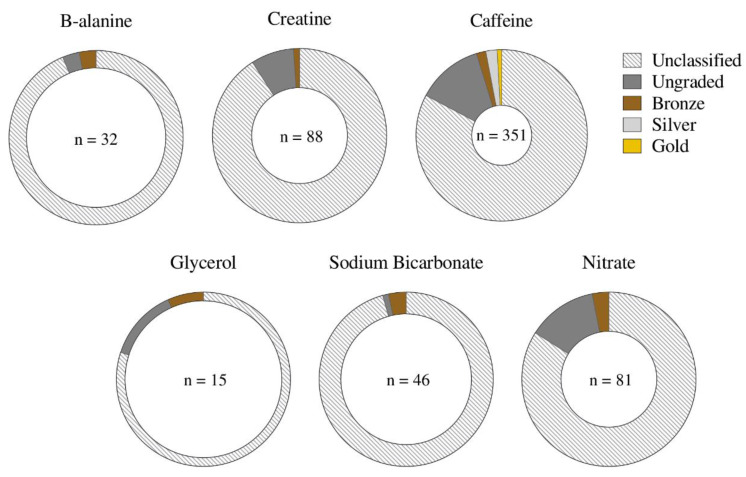
Proportion of studies classified into each tier [1], reflecting the standard of methodological control regarding ovarian hormonal profiles, as a proportion of the total number of studies including women across each supplement. Gold standard (best practice methodologies, as outlined by Elliott-Sale et al. [27]), silver/bronze (achieve some, but not all, methodological considerations), ungraded (menstrual status is defined, but methodological control is insufficient to award bronze/silver/gold) or unclassified (insufficient information to provide a robust classification of participants, or a mixed female cohort in which individual menstrual status cannot be discerned).

Eighty-nine (14%) of the 614 studies including women attempted to define their menstrual status, identifying naturally menstruating women (62 studies), HC users (13 studies), females with menstrual irregularities (1 study), and a mixed, but identifiable, female cohort (13 studies). However, the classification methods were poor, with 65 studies being ungraded (i.e., attempted to classify menstrual status, but provided insufficient information to be relevant) and few achieving bronze (13 studies), silver (8 studies) or gold (3 studies) standard [1] (Figure 5). Five studies specifically investigated changes in the supplement response across the menstrual cycle (MC) [22,23,24,25,26], meeting silver (three studies) or bronze (two studies) standard. Although 5 out of 62 studies involving naturally menstruating females claimed their participants were eumenorrheic, none fulfilled the five criteria required to justify this classification [27]. Overall, studies on naturally menstruating women achieved a mean of 0.3 of these criteria, with the highest score (2 of 5 criteria) achieved by five studies.

### 3.4. Performance vs. Health Research Themes

**Figure 6:**
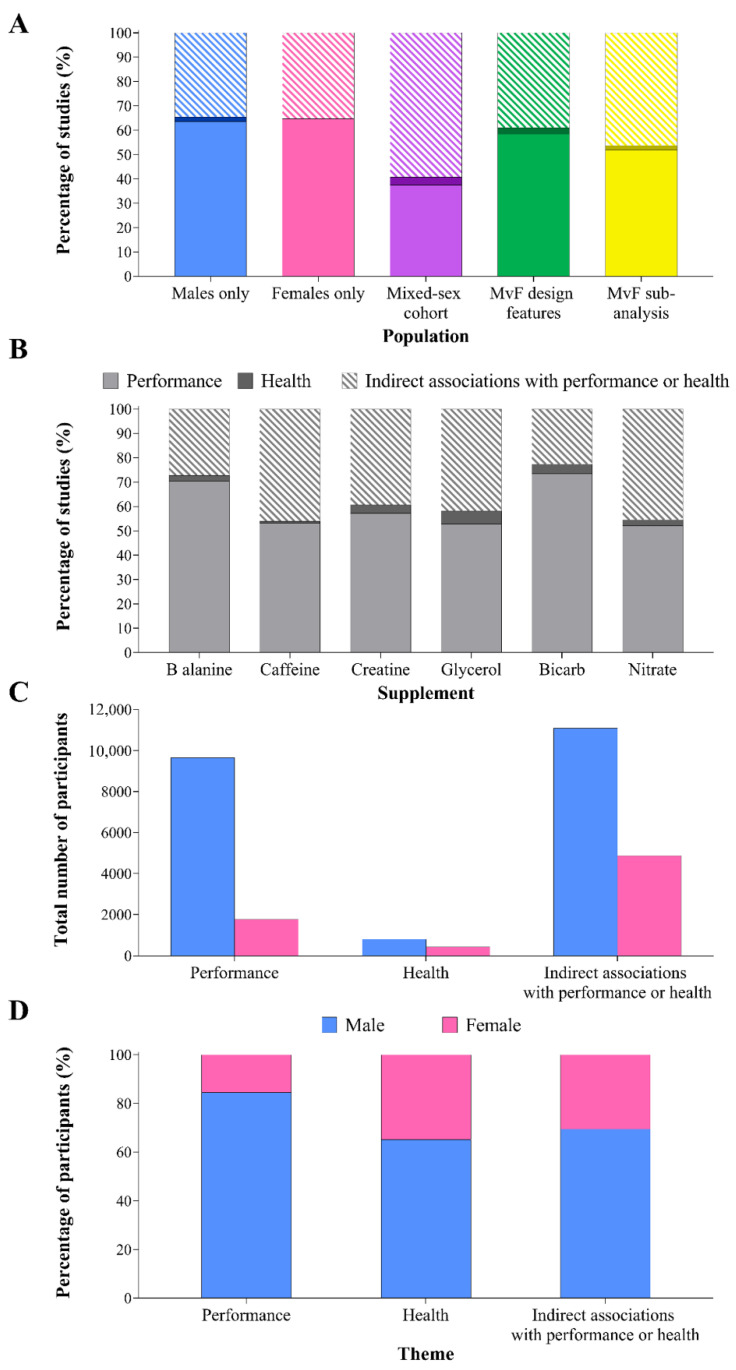
Percentage of studies in each theme, separated by (**A**) population (colours correspond to those used in Figure 3A) and (**B**) performance supplement. Number of participants represented as the (**C**) absolute number and (**D**) percentage of the total participants in each theme: performance (direct performance outcomes), health (outcomes related to health status/condition) and indirect associations with performance/health (a physiological or psychological adaptation/response that may subsequently transfer to athletic performance/health). Male versus female (MvF).

Across all supplements, performance was the most frequently investigated theme (57% of studies), with health outcomes and indirect markers of performance/health accounting for 2% and 41% of studies, respectively. Female-only studies were dominated by performance interests (54–100% of studies), with no studies targeting the health outcomes of any supplement (Figure 6A). Mixed cohort studies were most likely to investigate the indirect markers of performance/health (59% of studies). Studies of direct performance outcomes accounted for 70% (*n* = 88) and 73% (*n* = 149) of studies in β-alanine and sodium bicarbonate, respectively (Figure 6B), but represented a smaller proportion of the investigations in other supplements.

Although studies measuring indirect associations with performance/health accounted for only 41% of all studies, these investigations involved the greatest total number of participants (15,971 individuals, including 4875 women, representing 56% of participants across all supplements, Figure 6C). Health-focussed studies involved the lowest absolute numbers of participants (1253 individuals). However, these studies had the greatest relative proportion of female participants (35%, 437 individuals), while performance studies displayed the poorest representation of women (16%, 1777 individuals, Figure 6D).

### 3.5. Journal and Study Impact

**Figure 7:**
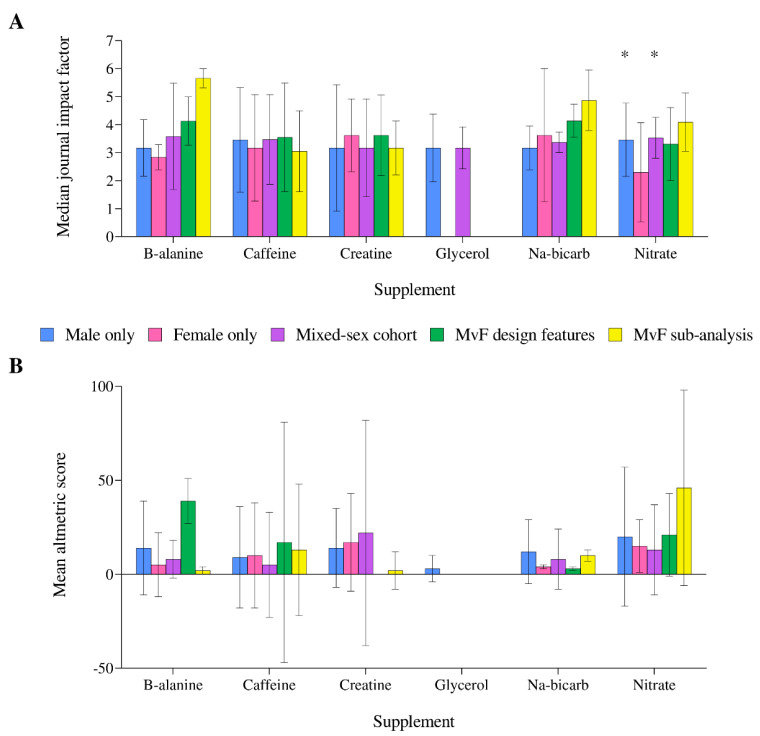
Median journal impact factor (**A**) and Altmetric score (**B**) across all performance supplements, separated by population studied. * Significantly greater impact factor compared to the nitrate studies in a female-only cohort (*p* < 0.01). Error bars display the interquartile range. Male versus female (MvF).

Across the different supplements, the median IF of journals in which studies were published ranged from 2.3–5.7. Regarding nitrate supplementation, studies in a female-only cohort were published in journals with a lower IF (2.30) than male-only (3.46, *p* = 0.005) and mixed-sex cohort (3.53, *p* = 0.002) studies (Figure 7A). Altmetric scores were only available for 56% of studies and were highly variable, with a median ± IQR score across all supplements and populations of 11 ± 27, and no differences between supplements or populations (all *p* > 0.05, Figure 7B). Studies involving male-only cohorts represented the highest number of publications (*n* = 255) with Altmetric scores >20 across all supplements. Studies utilising MvF design features had a greater proportion of publications with Altmetric scores >20 (*n* = 12 studies, 33%), comparative to other populations (<23%), but the low total number of studies (*n* = 33) must be considered. In terms of overall supplement interest, nitrate (*n* = 103, 43%), β-alanine (*n* = 36, 29%) and caffeine (*n* = 167, 20%) had the greatest proportion of studies with Altmetric scores >20.

## 4. Discussion

We used a recently developed tool [1] to undertake a literature audit of the representation of female and male participants in investigations of credible performance supplements (β-alanine, caffeine, creatine, glycerol, nitrate/beetroot juice and sodium bicarbonate). Our objective was not to examine the effectiveness of each product, but to scrutinise the quality and quantity of research pertaining to female athletes, particularly high-performance competitors. There were marked differences in the size of the literature supporting the use of each supplement, ranging from 55 investigations of glycerol to 858 studies of caffeine. Females accounted for just 23% of the total participant count, while the total number of studies targeting females exclusively was ~12 times fewer than those only involving male participants. Nevertheless, females who participated in these studies were more likely to represent higher calibre athletes. However, 99.5% of studies including female participants involved an inadequate methodological design around the categorisation and standardisation of menstrual status. We conclude that even though these supplements are identified by expert groups as evidence-based products that increase athletic performance [15], specific support for their use by female athletes is lacking in quantity and quality.

### 4.1. Population and Sample Size

Overall, women accounted for 23% of the total participants in the performance supplement literature. This appears lower than the 34–42% representation previously reported in other SSSM audits [2,3,4] but may be explained by differences in the topics and source for our audits. Whereas others have audited numerous topics within a defined set of SSSM journals over a specified period [2,3,4], we examined all published literature (~50 years) on a narrow range of topics across all available SSSM journals. Indeed, our findings are similar to the 21% representation identified in an audit of a specific SSSM topic across all journals [5]. The low total involvement of female participants in the present audit reflects both the lower number of studies involving female-only cohorts, as well as lower numbers of women within the sample among the 14–33% of studies with mixed-sex cohorts. A few studies failed to directly state the sex of the participants, reinforcing the notion that male research has traditionally been considered the norm.

Investigations on exclusively female cohorts accounted for 0–8% of studies across different performance supplements, similar to the 4–13% previously reported across a range of exercise/sports themes [2,3,4,5,6]. There is no evidence that the attention drawn to this inequity in research participation [3] has yet translated to changes in practice, since the proportion of female-only studies published over the previous 5 and 10 years (0–7% and 0–8%, respectively) is similar to the overall time period. Studies involving male-only cohorts in our audit accounted for a greater proportion of total studies (59–77%) than the 18–39% previously reported [2,3,4,6], but resembles the 70% reported in the only other topic-specific sports science audit (exercise thermoregulation) [5]. This difference reflects a lower proportion of mixed-sex cohort studies in our audit (14–29%) compared to previous work (35–78%) [2,3,4,6], again echoing different topics and timespans. Unfortunately, such studies provided the most likely opportunity for female inclusion in the audited literature. Indeed, across supplements, 56–100% of female participants were involved in mixed-sex designs where methodology ignores the potential influence of sexual dimorphisms on the study findings. Such protocols may be acceptable for themes where the absence of differing responses between men and women has been established. However, as this has not been sufficiently investigated in the performance supplement literature, a mixed-sex cohort could be considered inappropriate. Studies specifically designed to investigate sex differences in response to supplementation were the least prevalent model, representing 0–2% of all studies.

Although women accounted for 23% of the participant pool, they were included in 34% of all studies, indicating an unequal participation rate, even when eligible for research inclusion. Indeed, both mixed-sex and MvF designs involved greater numbers of males within the cohort. Such a discrepancy warrants investigation, with potential contributors including smaller numbers of women within teams/clubs or professional sports per se [28], difficulties in recruiting female participants (e.g., volunteer bias) or a lack of funding/resources to facilitate the additional requirements for undertaking research on female athletes (e.g., menstrual classification/control). Smaller sample sizes are problematic in various types of supplement research, challenging the robustness of the MvF design or sub-analysis protocols in general, as well as parallel group designed projects, which are needed for products requiring lengthy supplementation or withdrawal protocols (e.g., creatine/β-alanine supplementation).

### 4.2. Athletic Calibre and Research Theme

The participants in more than half of the audited studies were “unclassifiable” in terms of athletic calibre, highlighting the overall inadequacy of participant characterisation across the SSSM literature, regardless of sex. When classification was possible, most (65%) participants were placed in Tiers 1 and 2, while 70% of performance-themed studies were conducted in Tier 0–2 participants. This is a potentially incongruous population choice, despite its prominence in terms of the size of the consumer base, since many could possibly benefit more from other strategies (e.g., training) than supplement use. Logically, when higher calibre athletes (Tiers 3–5) were involved in studies, they were almost exclusively focused on performance outcomes (86–100% of studies). Nevertheless, the absolute number of participants of the highest athletic calibre was low: only 9% (*n* = 778) of men and 19% (*n* = 323) of women across all performance-focussed studies were classified as Tiers 4 or 5. This is concerning, since competitive athletes likely gain the greatest rewards from achieving even marginal gains in performance but may also differ in their response to some supplements compared to recreationally trained individuals due to genetic or training-induced differences [29,30,31,32,33]. We noted differences in research themes and participant calibre between supplements; β-alanine and sodium bicarbonate had the greatest proportion of studies of performance and involvement of higher calibre athletes (Tiers 3–5), reflecting their sports-specific uses. Meanwhile, we noted a more versatile application of glycerol and caffeine studies to occupational exercise scenarios (e.g., defence, manual labour, firefighting).

Given the overall dominance of male participants and male-focussed study designs, it is intriguing to find a higher proportion of female participants among studies involving Tier 4 and 5 athletes. Most (71%) of these studies were published in the last 10 years, suggesting that elite female athletes have been more willing to volunteer in studies and/or have attracted research interest. Unfortunately, of the 38 studies investigating Tier 4 to 5 female athletes, only 6 attempted to characterise menstrual status (*n* = 1.5 studies bronze standard [34,35], *n* = 4.5 studies ungraded [36,37,38,39]), thus reducing the transferability of the findings to all cohorts of female athletes.

Nevertheless, the overall under-representation of women in research was most noticeable in studies measuring performance outcomes, in which 16% of the total participants were female. The higher representation of women in supplement studies measuring health (35%) or indirect associations with performance/health (31%) may reflect the comparative ease of recruiting less athletically trained women who may still be suited to these outcome measures. Paradoxically, although studies investigating exclusively women focussed on performance outcomes (65%, *n* = 66 female-only studies), the absolute number of these studies was ~12 times fewer than those in male-only cohorts. It may be that the more recent inclusion of women in SSSM research has focussed on verifying the performance benefits of these supplements established in males, with additional uses subsequently investigated. Similarly, a greater proportion of health studies in male-only research may be an artefact of the higher total study number.

### 4.3. Menstrual Status

Overall, the menstrual status classification and methodological control within the audited studies of female athletes was extremely poor. Of the 614 studies including women, 86% made no attempt to classify menstrual status, and only three studies (~0.5%) implemented the ‘gold standard’ [27] level of menstrual classification and methodological control; indeed, all three studies were from the same author group and studied HC users [40,41,42]. Concerningly, the characterisation of menstrual status and methodological control remained poor in the five studies investigating changes in the response to caffeine across the MC [22,23,24,25,26]. Although better than average (three silver, two bronze), they all failed to achieve the required level of methodological control to be considered gold standard [27], thus reducing confidence in the findings.

The performance supplement literature does not reflect the menstrual status of the female athlete population. Notably, we failed to find a single study conducted on women confirmed to be eumenorrheic, although five studies claimed to include this cohort. Since surveys report that ~50% of female athletes are not taking hormonal contraception (HC), and hence are eumenorrheic or with menstrual irregularities [43], this is clearly an area for future development. The 50% of the female athlete population utilising HC [43] are represented in a very small number of studies and there is almost a complete absence of research in women with menstrual irregularities (three studies), despite an estimated prevalence of menstrual disorders in athletic populations of 13–60% [44,45,46]. The challenges of recruiting a uniform and suitably large sample of participants with the various menstrual disorders (e.g., amenorrhea, oligomenorrhea, luteal phase deficiency) are acknowledged. These conditions also require confirmation from a medical doctor, potentially adding a logistical hurdle and additional step to the screening process. Moreover, the priority for these individuals should be treatment, rather than participation in experimental trials, which may also challenge study recruitment and delay individual treatment. Nevertheless, further research into individuals with menstrual irregularities is warranted. Within the current audit, 13 studies investigated a heterogeneous population of women (i.e., a combination of naturally menstruating/HC users/menstrual irregularities). A mixed population of women in a single cohort is of little use unless the study is designed to compare the response to an intervention between women with differing menstrual status, with each category correctly classified and appropriate methodological steps implemented. Failure to do so is likely to introduce heterogeneity in findings.

Taken together, only three studies (~0.5% out of 614 published supplement studies including women) used gold standard methodologies to assess menstrual status. Failure to achieve robust characterisation and methodological control of menstrual status introduces potential variability into the results, while also reducing the ecological validity and applicability of the findings to specific female athlete populations [27]. Therefore, the current supplement literature fails to provide meaningful and credible recommendations regarding the efficacy of performance supplements in female athletes; high-quality research across all performance supplements and among women with different menstrual characteristics is therefore urgently required.

### 4.4. Journal Publication Dates and Study Impact

The IF of journals supporting supplement publications appeared consistent between products and populations. The only difference was observed in female-only nitrate studies, which were published in lower IF journals than male-only and mixed-sex cohort studies of nitrate. However, this observation may reflect an interest in the supplement itself rather than a broader lack of interest in female participants. With this exception in mind, there does not appear to be any sex bias in the actual/perceived interest in, and value of, the findings between populations. Further, the propensity for the manipulation of IF to a journal’s advantage should also be considered when interpreting these findings [1].

The limited availability of Altmetric scores, alongside the large variability, limits any strong inferences from these data. Male-only study designs had the largest number of studies with an Altmetric score >20; however, this may be an artefact of the larger total study number rather than a greater interest in this population. Similarly, the higher proportion of studies achieving an Altmetric score >20 in MvF design features studies is also likely to be a result of the small total number of studies in this population. It must also be considered that studies with an older age of publication are disadvantaged with this metric. Four out of six supplements had an average year of publication prior to the initiation of the Altmetric score in 2012 (ranging from 2003–2010). There were also too few high-quality studies with regards to menstrual status classification/methodological control or studies specifically assessing sex differences in supplement response, to allow any link between more challenging experimental designs and any apparent reward in study impact to be examined. It may, therefore, be the case that a larger literature base is required before these scores are able to be meaningfully interpreted.

## 5. Conclusions

Our audit demonstrates the poor representation of female athletes in the performance supplement literature. This bias is compounded by the inadequate classification and control of menstrual status in 99.5% of studies. Consequently, the current literature fails to provide robust recommendations regarding the efficacy of credible performance supplements in female athletes. Further research among female cohorts, using high-quality methodological approaches, is therefore required, with particular attention to athletes of high athletic calibre (Tiers 3–5) as well as robust classification and methodological control of menstrual status according to best practice guidelines [27]. Only then can the effects of performance supplements known to meaningfully enhance performance among male athletes be validated in their female counterparts, providing true confidence in the expert guidelines around the use of these products.

## Data Availability

Data (metrics extracted for analysis across the 1826 studies) are available from the corresponding author (ella.smith@acu.edu.au) upon reasonable request. Reuse is permitted with appropriate reference to this paper.

## Supplementary Materials

The following supporting information can be downloaded at: https://www.mdpi.com/article/10.3390/nu14050953/s1, Figure S1: B-alanine flowchart. Male (M), female (F), menstruating (men), hormonal contraception (HC). Bold text denotes papers, non-bold typeface is used for studies. From section B onwards, all counts, and percentages refer to individual studies (not papers). ◊ Studies comparing tier 4 vs. 1; Figure S2: Caffeine flowchart. Male (M), female (F), menstruating (men), hormonal contraception (HC). Bold text denotes papers, non-bold typeface is used for studies. From section B onwards, all counts, and percentages refer to individual studies (not papers). * Studies comparing tier 2 vs. 1, ^ studies comparing tier 2 vs. 0, ◊ studies comparing tier 4 vs. 1; Figure S3: Creatine flowchart. Creatine kinase (CK), phosphocreatine (PCr), male (M), female (F), menstruating (men), hormonal contraception (HC). Bold text denotes papers, non-bold typeface is used for studies. From section B onwards, all counts, and percentages refer to individual studies (not papers); Figure S4: Glycerol flowchart. Male (M), female (F), menstruating (men), hormonal contraception (HC). Bold text denotes papers, non-bold typeface is used for studies. From section B onwards, all counts, and percentages refer to individual studies (not papers); Figure S5: Sodium bicarbonate flowchart. Male (M), female (F), menstruating (men), hormonal contraception (HC). Bold text denotes papers, non-bold typeface is used for studies. From section B onwards, all counts, and percentages refer to individual studies (not papers). + Studies comparing tier 4 vs. unclassified; Figure S6: Nitrate flowchart. Male (M), female (F), menstruating (men), hormonal contraception (HC). Bold text denotes papers, non-bold typeface is used for studies. From section B onwards, all counts, and percentages refer to individual studies (not papers). ~ Studies comparing tiers 4 vs. 2 vs. 1 vs. 0, † studies comparing tiers 2 vs. 1 vs. 0, ᶿ studies comparing tiers 5 vs. 3 vs. 1.
