# Supplementary figures and images for: Auditing the Representation of Female Versus Male Athletes in Sports Science and Sports Medicine Research: Evidence-Based Performance Supplements
Source: Nutrients. 2022 Feb 23;14(5):953. doi: 10.3390/nu14050953 (PMC8912470; doi:10.3390/nu14050953)

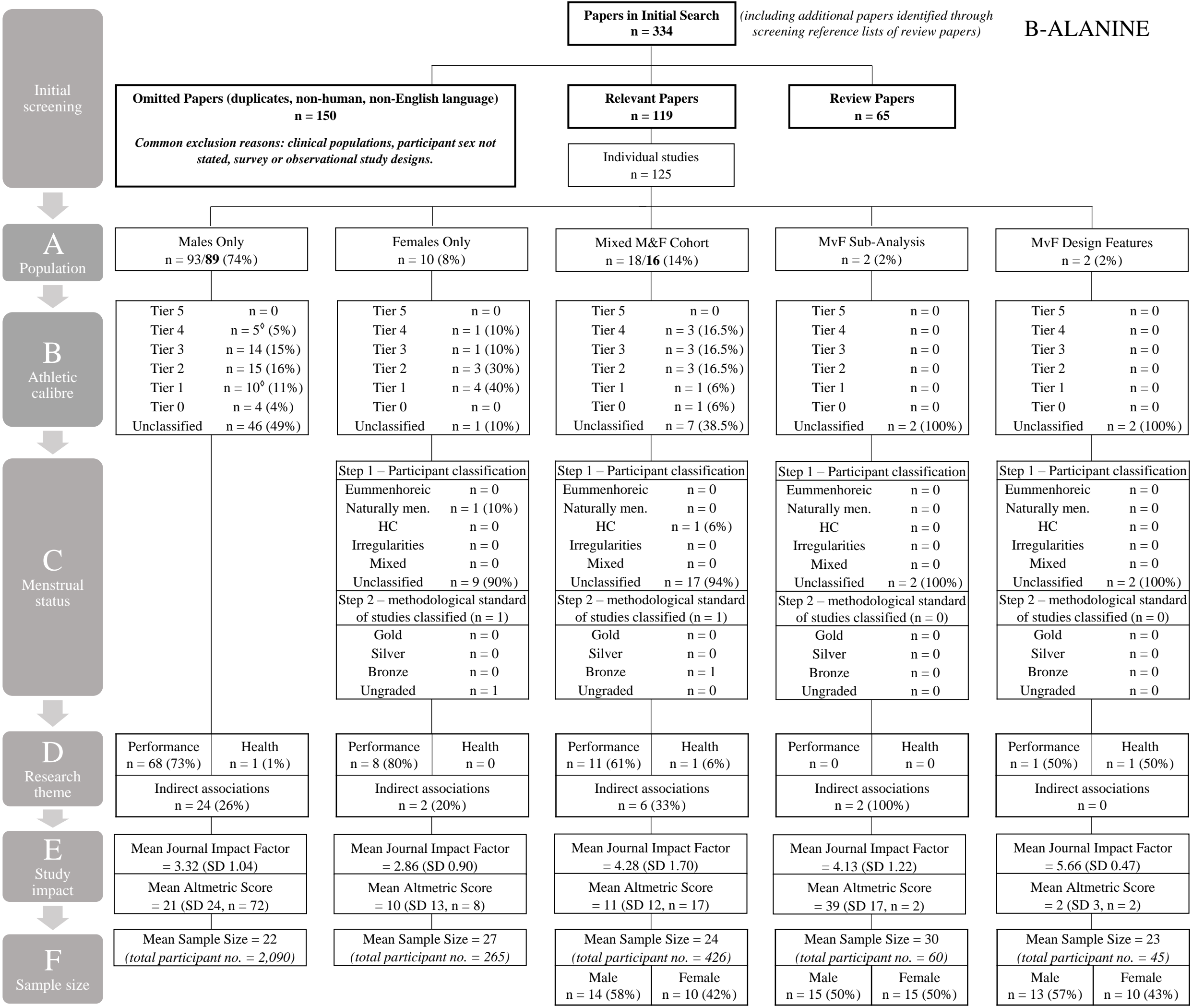

Supplement: Supplementary file 1 [file nutrients-14-00953-s001.zip › nutrients-1596830-supplementary.pdf]
